# Prick by Prick Induced Anaphylaxis in a Patient with Peanuts and Lupine Allergy: Awareness of Risks and Role of Component Resolved Diagnosis

**DOI:** 10.1155/2014/892394

**Published:** 2014-11-18

**Authors:** Anna Ciccarelli, Claudia Calabrò, Clara Imperatore, Guglielmo Scala

**Affiliations:** Allergy Unit, Loreto Crispi Hospital, Via Schipa 9, 80122 Naples, Italy

## Abstract

A case of anaphylaxis is reported in the course of a prick by prick with Lupinus albus and roasted peanut in a 20-year-old woman. We focused on some main topics. First of all it seems important to underscore the potential risks connected to the practice of the prick-by-prick with fresh foods in allergic patients, especially when testing cross-reactive substances, such as White Lupine, peanuts, or soy. It is important that clinicians who perform prick tests be aware of the risk related with in vivo tests in allergic patients. Second, we discuss the problem of the hidden allergens, such as White Lupine flour, or soy flour which are utilized to improve wheat flour because of their lower cost. Patients with a demonstrated allergy to peanuts should be assessed for lupine allergy and informed about the “hidden allergens” issue. Finally, we believe that component resolved diagnosis, the serum specific IgE against molecular components, that is normally considered a second-level diagnostic step has an important role even as a first line approach at least in some selected cases.

## 1. Introduction

Skin prick test (SPT) is the most widely used first level diagnostic test for IgE-mediated allergy. The test that evaluates the presence of allergen-specific IgE at skin level is fairly inexpensive and gives results in few minutes. As compared to the dosage of serum-specific IgE the prick test has several advantages. SPTs are quick, are simple to perform, and are pedagogical too, as the result is immediately obvious to the patient's eyes. The outcome is dependent on several parameters such as the extracts used, the drug assumption, the conditions of the skin, such as the presence of dermographismus, the clinician's experience, and the lancet utilized. The possibility is well known of getting false negative or false positive results. Specified rules for allergy testing, including SPTs, have recently been recommended for children [1]. Since it is an “in vivo” test, the most important point to consider is the safety. Even though skin prick testing is mostly safe, systemic reactions have so far been described especially when fresh food is utilized in the so-called prick-by-prick technique [2]. Because of the complexity of interpretation and for the theoretical possibility of adverse reactions, prick tests should be carried out by trained health professionals [1]. Actually clinicians may perform prick by prick without having been trained by specialists and without having emergency experience.

Lupine (*Lupinus albus*, White Lupine), peanut, and soy are members of the Legume family, the second largest family of seed plants. Lupine flour may be considered as a classic “hidden allergen.” It has been found to be included at a level of 10% in wheat flour without mandatory labelling [3] and lupine-fortified pasta has been found responsible for allergic reactions [4]. Furthermore it is now being used as an alternative to soy flour by companies seeking nongenetically modified food ingredients [5]. Legumes are well known as allergens but lupine allergy alone is still rarely reported [6]. Sensitization can occur through the oral route but also through inhalation. No general consensus exists so far on the allergic composition of Lupine. Lupine allergy has been mainly reported in patients with allergies to other legumes, particularly peanut [7]. A serologic cross-reactivity with soy and peanut is common [8] and sequence homologies have been detected among lupine Lup a gamma-conglutine and peanut Ara h1 viciline and Ara h3 glicinine [9]. A further homology has been identified between Ara h 8 and the lupine PR-10 (pathogenesis-related protein) both being Bet v 1-related molecules (http://www.allergome.org/). Recently a Lipid Transfer Protein (LTP) has been identified in peanuts, which cross-reacts with Pru p 3, the peach LTP and the lupine Lup a LTP [10, 11].

We describe a case in which a young woman had a quite serious systemic reaction while performing a prick by prick with roasted peanut and lupine.

## 2. Case Report

MTL, 20-year-old woman, was referred to our clinic for suspected peanut allergy. Three weeks before we met she had presented oral itching and generalized urticaria after she ate few peanuts. The skin reaction had started twenty minutes after the contact with the allergen without other respiratory or gastrointestinal symptoms. She had immediately taken cetirizine and betamethasone and was well in one hour without further complications. During childhood she had been diagnosed with peach allergy. A skin prick test (SPT) was performed with dust mites, grass, pellitory, olive tree, cat,* Alternaria*, latex, birch,* Artemisia*, peanut, chestnut, hazelnut, almond, and soya plus histamine and saline solution (all extracts by Stallergenes, Antony, France). In addiction we performed on the other forearm a prick by prick (PbP) with roasted peanut and boiled lupine. A 1 mm single peak lancet (ALK-lancet, Copenhagen, Denmark) was utilized. The patient was not taking antihistamines.

The peanut extract and the peanut and lupine PbP showed positive result in 5 minutes, with a 5 mm wheal. In the following minutes the peanut and lupine PbP wheals surpassed the diameter of 20 mm, eventually conflating. Ten minute after the prick test, a severe oedema of the interested arm appeared, with intense itching followed by the spread of urticaria. The patient was transferred to the emergency room and a vein line was obtained. Shortly after, dry cough, dyspnoea, bronchoconstriction, and conjunctival erythema started. The patient was given intramuscular epinephrine, intravenous fluids, hydrocortisone, and promethazine. Two hours later, a blood sample was taken for specific IgE, tryptase, and other routine exams. In the next thirty minutes the skin reactions began to diminish, eventually disappearing. The cough subsided and finally the throat restriction and the dyspnoea disappeared. Blood pressure was normal. The patient was kept under observation until the next morning and prescribed self-administrable epinephrine. Laboratory results were as follows: tryptase 18 mcg/L, specific IgE (ImmunoCAP Phadia, n.v.: < 0.10 kUA/L): peanuts 28.12, Pru p 3: 12.40, Ara h1: 12.12, Ara h9: 7.20. Notably Ara h9 and Pru p3 are the LTP component, respectively, of peanut and peach and both cross-react with the Lup a LTP which is the LTP component of lupine (see below).

## 3. Discussion

We describe a case of anaphylaxis caused by a PbP with peanut and lupine. What makes this case particular is that we were aware of the peanut and peach allergy, likely due to a LTP sensitization, but we did not actually expect the dramatic experience we went through by doing the two tests for peanut and lupine together. Our experience has some points of interest. We do confirm the well-known possibility of cross-reactions among Leguminosae with the potential risk of anaphylaxis but the relevance of our case lies elsewhere. Adverse reactions to SPT and PbP have been reported but an emergency treatment equipment is not required in general practice [11]. In our case things could have been worse if we had not had easy access to an emergency treatment. Nowadays there are more and more untrained clinicians who perform prick test on their patients. It is important that all physicians who deal with allergy be aware of the risk of the prick-by-prick technique with fresh foods, even when performed with allergens unlikely to be dangerous.

Finally, although there have been an ever increasing number of reported cases of lupine allergy, White Lupine allergy is still largely overlooked by clinicians. There is a great need for the education of food-service workers and the allergic consumer on the practices of the food-service industry with regard to “hidden” allergens, as this is the area in which many severe and fatal reactions are reported.

It seems reasonable that patients with a known allergy to peanuts should be advised to avoid all products containing lupine until they can be specifically tested.
